# Apparent increase in lip size influences two-point discrimination

**DOI:** 10.1038/s41598-023-30067-3

**Published:** 2023-02-22

**Authors:** Elisabetta Ambron, H. Branch Coslett

**Affiliations:** 1 Laboratory for Cognition and Neural Stimulation, Perelman School of Medicine at the University of Pennsylvania, 3710 Hamilton Walk, Philadelphia, PA 19104 USA; 2 Department of Neurology, Perelman School of Medicine at the University of Pennsylvania, Philadelphia, USA

**Keywords:** Perception, Human behaviour

## Abstract

Magnified vision of one’s body part has been shown to improve tactile discrimination. We used an anesthetic cream (AC) to determine if somesthetic stimulation that alters the perception of the size of one’s body would also improve two point-discrimination (2PD). In Experiment 1, application of AC caused an increase in perceived lip size and an improvement in a 2PD. As perceived lip size increased, subjects became more accurate in identifying that they had been touched in two locations. Experiment 2 confirmed this effect in a larger sample and introduced a control condition (no AC) that demonstrated that the change in performance was not attributable to practice or familiarity with the task. In Experiment 3, we showed that both AC and moisturizing cream improved subjects’ ability to indicate that they had been touched in 2 locations, but the improvement was modulated by perceived lip size only for AC. These results support the idea that changes in the body representation influence 2PD.

## Introduction

Multiple sources of information, including sensory and vestibular inputs as well as feedback from action, are integrated to generate a representation of the body that specifies the position and size of body parts^1–3^. Evidence for the dynamic, multisensory nature of this representation comes from demonstrations that the perceived size of a body part can be increased by modifying visual^3–7^ or somatosensory inputs^2,8^.

Several investigators have demonstrated that changes in the perceived size of a body part may have consequences for sensory^4^ or motor^6^ functions. For instance, Kennett et al.^4^ showed that performance on a two-point discrimination (2PD) task involving the forearm improved when looking at the forearm through magnifying lenses as compared to normal vision (magnification effect). Taylor-Clark et al.^7^ showed that the magnification of a body part using vision increased the perception of the distance between two points stimulated on the skin. Additionally, magnifying lenses improved motor function in subjects with stroke^10^ and increased motor cortex excitability in normal subjects^11^. Indeed, using transcranial magnetic stimulation, we showed that motor-evoked potentials recorded from the hand area of the motor cortex increased when participants were looking at their hand through magnifying glasses as compared to normal vision. This increase occurred not only with TMS stimulation at the motor “hot spot” (the area in which the motor threshold is reached with the lowest TMS stimulation) but in surrounding areas of the motor cortex, suggesting that magnification may induce a rapid remapping of the magnified area in the primary somatosensory and motor cortices^11^.

Changes in the perceived body size have not only been explored through vision but also via somatosensory inputs. Some examples of somatosensory manipulations are the application of a tendon vibration to induce the sensation of a longer arm^13^ or a longer nose (so-called the Pinocchio illusion)^14^. Additionally, Canzoneri et al.^15^ demonstrated that the prolonged use of a long tool that changed the perceived length of participants’ forearm resulted in an apparent increase in the subjects’ representation of arm length to match the body size with the inclusion of the tool^15^.

Although previous work has explored the effects of visual perturbations of body size on sensory and motor function, the effects of magnification of a body part achieved by non-visual manipulations have been less extensively explored. Using tendon vibration, D’Amour et al.^16^ induced the illusion of expansion or shrinkage of the arm and waist and showed a reduction in both tactile acuity and detection when the body representation was altered. The authors argued that these changes in the body representation would alter the mapping of the tactile stimuli and generated noise in tactile processing. It should be noted that these results are inconsistent with previous studies involving visual manipulation^4,7^. If the effect of magnification induced by tendon vibration were the same as those induced by visual input^4,7^, one would predict that improved tactile discrimination would be observed when body parts are perceived as larger. Thus, the effects reported by D’Amour et al.^16^ raise the possibility that magnification via changes of somatosensory input be mediated by different mechanisms than are at play with visual input or may be specific to effects induced by tendon vibration.

To test the effect on the tactile sensation of magnification of a body part via somatosensory input, we exploited the observation by Gandevia and Phegan^8^ that the application of an anesthetic cream (AC) to the lips was associated with an increase in perceived lip size without complete anesthetization of the lips. We replicated this finding in pilot testing (n = 4) using a different anesthetic (benzocaine); we found that participants experienced an increase in lip size while retaining the ability to perform two-point discrimination (2PD). In Experiment 1 we presented individuals with 2PD on the lips in three conditions: before application of the AC, shortly after the AC was applied (when the lips should be perceived as enlarged), and after the AC was removed and perceived lip size returned close to normal. Full anesthetization of the lips was not induced, but the application of the cream was enough to induce the sensation of an increase in lip size in most participants. If local anesthetics induce the perception that the treated body part is larger, and this representation of the body underlies performance on a 2PD, one would expect subjects to be more accurate after the application of the AC if they perceive an increase in lip size as an effect of the AC. Based on the evidence that the visual magnification of a body part increases the perceived distance between two points on the body surface^7^, we predicted that participants would be more likely to distinguish between the touch of 1 or 2 points after the application of the AC; furthermore, we predicted that this effect would be most pronounced in trials with the shorter inter-stimulus distances (e.g., 1 and 2 mm) as the incremental gain from the size manipulation would be more meaningful in conditions of greater uncertainty. Experiment 2 was undertaken to replicate the results in a larger sample and included a control condition to test the hypothesis that the improvement was related to learning and familiarization with the task. In Experiment 3, we tested whether the results were due to a non-specific effect of the cream; to that end, we contrasted the effect of the AC and a moisturizing cream (MC); MC has been demonstrated to improve 2PD^17^. Improvement of 2PD with the AC at short inter-stimulus distances (1 or 2 mm) was confirmed across experiments. The effects are not due to familiarization or learning (Experiment 2). Experiment 3 demonstrated that the application of a MC induced improvement of 2PD but subjects’ performance in 2PD improved as a function of the perceived increase in the lips size only with the AC, suggesting that different mechanisms may account for the performance in these two conditions.

## Materials and methods Experiment 1

### Participants

Nineteen adults (mean age = 23.4, *SD* = 4.9; 12 females) took part in the present study. The Institutional Review Board of the University of Pennsylvania approved the present study, including all the experiments (Experiment 1–3) reported in the present manuscript. Experiments were carried out in accordance with the Declaration of Helsinki and participants provided informed consent in all the experiments (Experiment 1–3) reported in the present manuscript.

Due to the novelty of our paradigm, the effect size could not be directly estimated based on previous work. However, we noted that studies exploring the ability to perceive two points stimuli on different segments of a face^18^ or testing how changes with the body schema modulated two-point discrimination^19^ reported large effect sizes (~ *d* = 1.2). Power analysis was conducted with sjstats package in R to test the effect of changes in the identification of 2 points across distances (0, 1, 2, 3 mm) and time points (PRE, EXPERIMENTAL, POST). The analysis showed with at least twelve participants we had a power of 80% (*p* < 0.05) to detect a large (*d* = 1.2) effect size.

### Task

Subjects were tested with a two-point discrimination task (2PD)^5^ in which they were asked to determine if the lower lip was touched with one or two points. During the task, participants closed their eyes. The task was performed in three conditions during one session lasting approximately 60 min: (1) prior to application of AC (PRE condition), (2) ~ 2 min after application of AC on both upper and lower lip across the entire lip surface (Lanacane, benzethonium chloride 0.2% and 20% benzocaine) (EXPERIMENTAL condition), and (3) ~ 2 min after the AC was removed (POST condition). The cream was squeezed directly from the tube covering ~ 5 cm of the participants’ index fingertip. Participants were then instructed to apply as much cream as possible on both lips. This process was monitored by research personnel.

In each condition, 90 trials were performed for a total of 270 trials. On 45 trials, the distance between the two calipers was 0 mm; on the other 45 trials, subjects were touched with two points spaced at a distance of 1, 2 or 3 mm (15 trials each). In each of the three conditions, these stimuli were applied manually at the center of the lower lip using a two-point discriminator (Baseline 12-140 Aesthesiometer, Fabrication). On each trial, subjects were instructed to indicate if their lips were touched at one or two points by depressing the appropriate key on a button box. The task was programmed using E-Prime 3.0 software (Psychology Software Tools, Pittsburgh, PA) to randomize the order of the stimuli and of the response keys. Ninety trials were completed in approximately 15 min.

Subjects’ perception of their lip size was also assessed before each of the 3 conditions using two measures in which images of lips of increasing size were presented alone (lips only measure) or within a facial frame (whole face measure). For the lips only measure, ten photographs of Caucasian women’s lips of increased size (1 mm increase) were vertically arranged from the lower (13 × 35 mm) to the higher (23 × 35 mm) size, and participants indicated the image of the lips that best represented the perceived size of their lips at that moment in time. For the whole face measure, participants were shown 8 drawings of a woman’s face silhouette (25 × 30 mm) on which the lips were depicted in sizes ranging from 5 × 2 mm to a maximum of 7 × 5.5 mm, with a vertical increase of about 0.5 mm, arranged in a 2 × 4 array from small to large size; subjects were asked to point to the face that depicted the size of their lips at that moment in time. Although similar, these measures are different in the naturalistic aspects (drawing or photos), whether the lips were represented alone or within a framework, and in the range of responses (eight or ten). As these elements may have differentially facilitated participants in their judgments regarding their lips size, we decided to test and report the effect of both measures independently.

### Data analyses

Data analysis focused on three main research questions. First, we tested whether the application of AC induced an increase in perceived lip size. A Friedman test with time points (PRE, EXPERIMENTAL, POST) as a factor, was run for each lip size rating and numbness. Bonferroni corrected Wilcoxon post-hoc test was implemented (pairwise.wilcox.test function) in R to obtain the statistical significance of the difference across timepoints and the effects sizes (r value) were estimated using the wilcox_effsize function. Bayesian statistic was used to further explore non-significant differences and implemented with BayesFactor package in R. Bayesian Factor (BF10) < 1 would support the null hypothesis (1–0.33 anecdotal; 0.33–0.10 substantial; 0.10–0.03 strong; 0.03–0.01 very strong; < 0.01 decisive), while BF10 > 1 would support the alternative hypothesis^20,21^.

Second, we explored whether the proportion of yes responses (indicating that two points were detected) increased with the application of AC in conditions of uncertainty (1–2 mm) using a logistic mixed effect model (LMM) in R (3.3.0) with subjects included as random intercepts, and time points (PRE, EXPERIMENTAL, POST) and distances (0, 1, 2, 3 mm) as categorical factors. These factors were inserted into the model sequentially and ANOVAs were used to test the difference between models with or without the inclusion of each factor. Only factors contributing significantly to the model fit were included in the final model. The omnibus of the model was further estimated using the pamer.fnc function in R. We opted for this type of analysis because it accounts for the variability in the performance across individuals^22^ and reduces the possibility of Type I error^23^. For LMM analyses, we estimated the effect sizes using the pseudo R2 for the overall model^24^ and applied R2 Cohen^25^ criteria to identify the effect size. In addition, we measured the effect size for specific model parameters semi-partial R2^26^. To test, whether possible differences were due to a response bias, we computed the response criterion^27^. A liberal criterion (negative criterion score) would indicate that the application of the AC increases the likelihood of participants responding yes when uncertain. Alternatively, a positive score indicates a more conservative bias with a tendency to respond ‘no’ to minimize possible false alarms.

Third, we tested whether 2PD performance with the AC depended upon the perceived changes in the lips size induced with the application of the AC. To this end, we computed the difference between the rating of perceived lip size in the EXPERIMENTAL as compared to the PRE condition. This difference was also computed for the POST condition to test whether the effects were specific for the EXPERIMENTAL condition or carried over in the POST condition. For example, if a subject rated their lip size as corresponding to the 3rd face on the eight-point scale in the PRE condition and the 5th face for the EXPERIMENTAL condition, the difference score was + 2. Next, we ran two LMMs for number of *yes* responses on two points stimulations for the EXPERIMENTAL condition with change in perceived lip size as continuous and distance (0, 1, 2 and 3 mm) as categorical fixed factors.

One participant who performed 2 SD below the mean of the overall sample in the PRE condition was excluded from the analyses. As our hypotheses and comparisons were *a prior*i specified, analyses were not adjusted for multiple comparisons.

## Results Experiment 1

**Figure 1:**
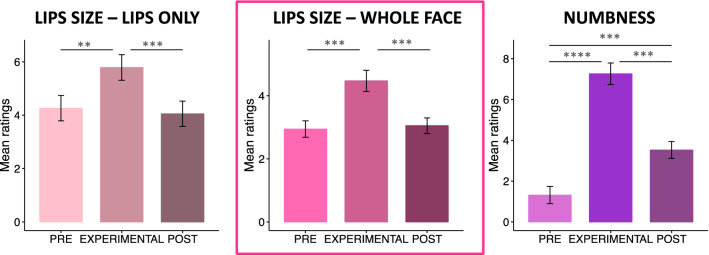
Mean rating scores for the lips size (lips only and whole face ratings) and numbness across time points (PRE, EXPERIMENTAL, POST) of Experiment 1. The error bars indicate the standard error of the mean. **p < 0.01; ***p < 0.005, ****p < 0.001.

First, we tested whether AC was effective in inducing a change in the perceived lip size and numbness (see Fig. 1). Friedman test confirmed a significant change across time points for all our measures (lips only χ^2^ = 17.44, *p* < 0.001; whole face χ^2^ = 20.77, *p* < 0.001; numbness χ^2^ = 29.51, *p* < 0.001). The perceived lip size increased with the cream application (lips only *p* < 0.01, *r* = 0.32; whole face *p* < 0.005, *r* = 0.49) and decreased once the cream was removed (lips only *p* < 0.01, *r* = 0.32; whole face *p* < 0.005, *r* = 0.49), so that participants’ ratings of the lip size in the POST were not statistically different from the PRE (*p* = 1, *r* = 0.05 in both ratings) condition. This last piece of evidence was partially supported by Bayesian analyses, that provide BF10 = 0.35 [anecdotal evidence for the null hypothesis] for the lips only and BF10 = 0.29 [substantial evidence for the null hypothesis] for the whole face rating.

**Figure 2:**
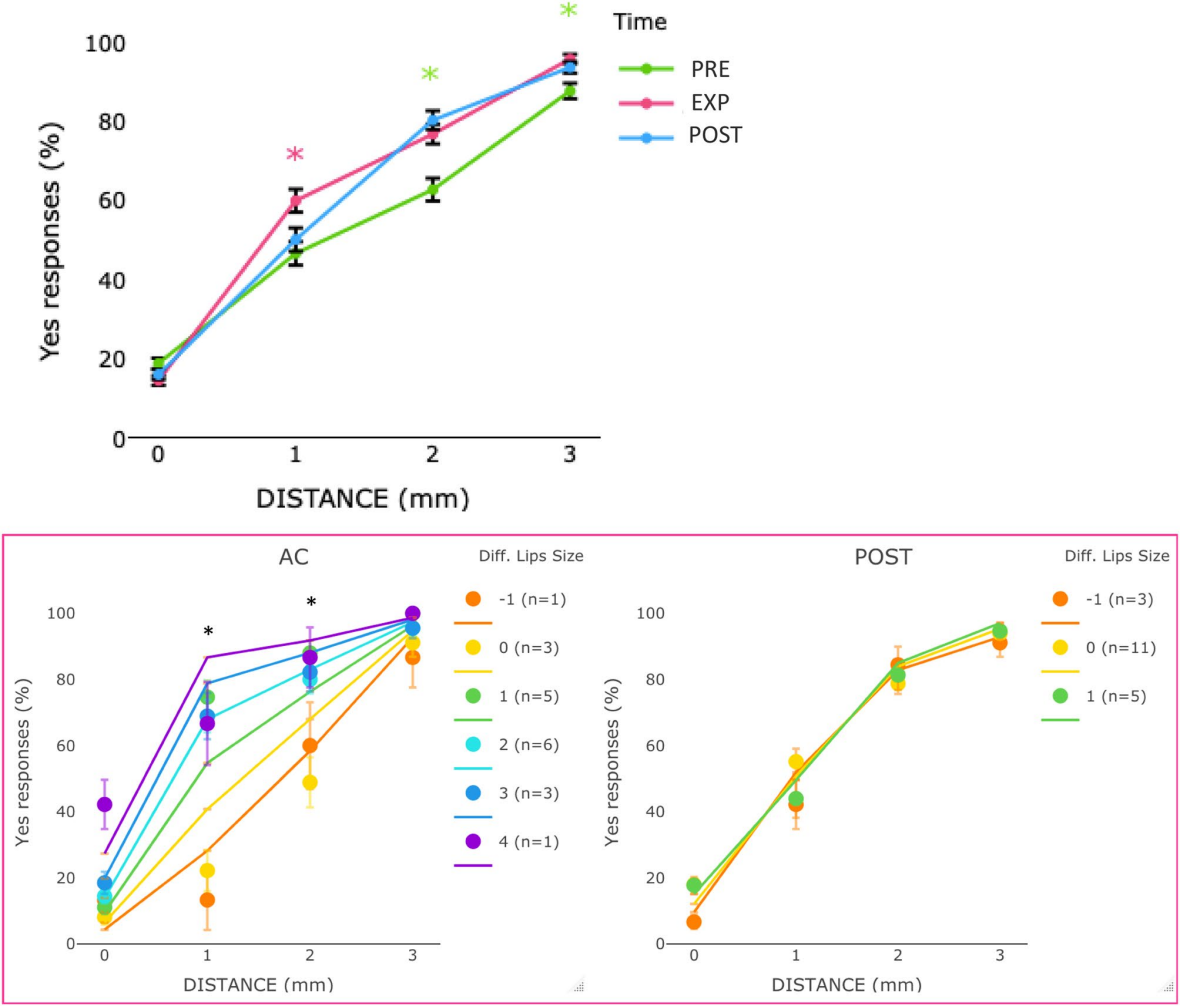
Top panel, mean (markers) and SE of the means (error bars) of *yes* responses in 2PD in PRE, EXPERIMENTAL and POST conditions for each distance (0, 1, 2, 3 mm) in Experiment 1. The star indicates significant (*p* < 0.05) effect. The color of the star indicates which condition differed from the others. Bottom panel, mean and SE of the means (error bars) of *yes* responses in 2PD in EXPERIMENTAL (left panel) and POST (right panel) conditions as a function of distance (0, 1, 2, 3 mm) between 2 stimulation sites and the change in the perceived lip size in Experiment 1. The legend describes the amount of perceived change in the lip size and the number of individuals who perceived that change. Negative numbers indicate a perceived decrease and positive numbers indicate an increase in the perceived lip size compared to PRE. For example, ‘− 1 (n = 1)’ indicates that one participant perceived a decrease in lip size of 1 unit, while ‘1 (n = 5)’ indicates that five participants experienced an increase in lip size of 1 unit. In the bottom row plots, colored lines represent the estimated effects in the LMM analyses and the markers the performance of individuals in the EXPERIMENT or POST based on change in the perceived lip size in that condition with respect to the PRE.

Second, we tested whether the application of the AC increased the detection of stimulation at two points in conditions of higher uncertainty. LMM showed a contribution of distance and time (logLik = − 2337, χ^2^ (8) = 58.1, *p* < 0.001, pseudo R2 = 0.50; see Table S1 in Supplementary Materials for more detail). In line with our predictions, participants detected stimulation at two-points more frequently in the EXPERIMENTAL as compared to the PRE condition when the distance was 1 (*z* = − 3.3, *p* < 0.001) and 2 mm (*z* = − 3.7, *p* < 0.001); participants were also more likely to respond yes in the 3 mm condition (*z* = − 3.4, *p* < 0.001; see Fig. 2). Performance was worse at 0 mm distance in the AC than PRE (*z* = 2.4, *p* = 0.01). Similar performance was observed in the AC and POST across all distances, except for 1 mm (*z* = − 2.4, *p* = 0.01).

To test whether these results were due to a response criterion rather than a change in sensitivity, we tested whether participants exhibited a more liberal criterion with the application of the AC. The results showed a change in the criterion with the application of the AC at a 3 mm distance condition (see Supplementary Materials). However, participants exhibited a more conservative criterion when the cream was applied (*M* = 0.17, *SE* = 0.02; Bonferroni’s adjusted *p* = 0.02) compared to the PRE (*M* = 0.06, *SE* = 0.02) condition.

Third, we tested whether the performance in the AC condition was related to changes in the perceived lip size. Following the results of the whole face measure (see Supplementary for lips only measure). To control for the possible influence of basic tactile discrimination ability, we entered the 2PD performance in the PRE condition in the model but it did not contribute significantly to the model and was removed. For the EXPERIMENTAL condition, the final model included both factors: distance and change in perceived lip size—whole face measure (logLik = − 668, χ^2^ (4) = 9.2, *p* = 0.05; pseudo R2 = 0.57). The main effects of both distance and lip size were significant (see Table S1 in Supplementary materials for the omnibus of the effects). Then we tested our prediction that the increase in the perceived lip size induced changes in the *yes* responses when there was a separation between the two points of 1 and/or 2 mm. We observed a significant increase in *yes* responses as a function of the increase in the perceived lip size for 1 mm (*z* = 3.1, *p* = 0.001) and 2 mm (*z* = 2.7, *p* = 0.02) distances (see Fig. 2 bottom-left panel). When the cream was removed and the sensation of increased lip size disappeared in the POST condition (see Fig. 1), the distance was the only significant predictor of *yes* responses (logLik = − 707, χ^2^ (3) = 871, *p* < 0.001; pseudo R2 = 0.54) (see Table S1 and Fig. 2 bottom-right panel).

## Comments on Experiment 1

A local anesthetic cream applied to participants’ lips increased the proportion of trials on which participants detected the touch of 2 points on a 2PD task; the tendency to report two touches (*yes* responses) was associated with the sensation that one’s lips had increased in size. This evidence is in line with literature on vision showing that the magnification of a body part improves 2PD^4^ and increases the perceived distance between two tactile stimuli^7^. Our findings demonstrate that the effect of the magnification of a body part on 2PD is not restricted to vision but can also be induced through somatosensory inputs. These effects were not accounted for by a general increase in yes responses with the application of the cream as participants showed a more conservative criterion for the 3 mm condition with the application of the cream.

An alternative explanation for the results of Experiment 1 is that the improvement in 2PD is due to learning effects and familiarization with the 2PD task across multiple testing sessions. Indeed, yes responses increased with AC, but performance remained stable for all distances except for 1 mm in the POST condition after the cream was removed. We explored this issue in Experiment 2 by introducing a control condition in which 2PD was tested on 3 occasions in the absence of AC. If the results of Experiment 1 are attributable to familiarization with the 2PD task, a similar learning pattern should be noted in conditions with and without AC. On the contrary, if the effects observed in Experiment 1 are related specifically to the AC, one would expect an increase of yes responses in 2 points stimulation conditions of uncertainty (1–2 mm distances) in the AC but not for the no AC condition.

Our results are surprising given that previous reports showed a decrease in tactile discrimination^28^ and a reduction of skin sensation^29^ with local anesthesia. How can we reconcile this apparent contradiction? First, some previous studies used an anesthetic cream (ELMA), which has been shown to be more effective in reducing tactile sensitivity compared to the AC creams that we employed^30^. Second, we tested tactile detection and 2PD a few minutes (~ 2 min) after the cream application, while in previous work testing occurred approximately 30 min after the application of the anesthetizing cream. It has been proposed that the decrease in tactile sensitivity may be the secondary effect of nociceptor blockade^30^ rather than a direct action of the local anesthetic on mechanoreceptors and cutaneous fibers. Indeed, benzocaine, a sodium channel blocker, primarily affects small, unmyelinated C-fibers responsible for pain sensation rather than large, myelinated fibers involved in touch and proprioception^31^.

It is possible that in our experiment, the type of cream used and/or the time between the cream application and task performance was not sufficient for the anesthetic to be fully effective^28,32^. Paqueron et al.^8^ showed that the effects of anesthetics on mechanoreceptors is maximal after 15–20 min; in our experiment, exposure to the AC lasted approximately 15 min. It is possible, therefore, that there was some effect from the AC on mechanoreceptors during the experimental session. In Experiment 2, we sought to formally evaluate the degree of anesthesia produced by the AC by obtaining measures of numbness using a rating scale and objective measures of tactile detection (Semmes–Weinstein monofilament, Stoelting).

## Methods Experiment 2

### Participants

Forty individuals (mean age = 21.8, *SD* = 3.4; 29 females) took part in this experiment. To test the effect of the fixed parameters condition (AC, no AC), distances (0, 1, 2, 3 mm), and time points (PRE, EXPERIMENTAL, POST). Power analysis with sjstats package showed that with a sample of at least fourteen subjects we had a power of 80% (*p* < 0.05) to detect a large (*d* = 1.2) effect size. We did not use the results of Experiment 1 to estimate the sample size of this experiment but increased the sample size to forty (twenty individuals in each group) to test whether the effects replicated in a larger sample of participants.

### Tasks

Each subject participated in two sessions. One session was the same as Experiment 1: 2PD measured in PRE, EXPERIMENTAL, and POST conditions. The other session had also the same structure (2PD measured in 3 time points), but no cream was applied in any condition. Other differences with Experiment 1 included the following: (i) 2PD was measured with an electric aesthesiometer (AOS ABSOLUTE Digimatic Caliper; resolution 0.01 mm); (ii) tactile detection threshold was assessed with Semmes–Weinstein monofilament (Stoelting) and subjective ratings of numbness were collected in the PRE, EXPERIMENTAL and POST condition in both conditions, using a visual analog scale from 0 (no numbness) to 10 (high numbness). The detection threshold was established by identifying the thinnest filament that the subject correctly detected on 2 of 3 trials. The procedure was initiated with a 5.8 mN filament and if they responded correctly on 2 of 3 trials, the next thinnest filament was presented.

### Data analysis

Two analyses were performed. First, we tested whether the AC application induced changes in tactile detection and numbness and whether similar effects were observed without the AC. To this end, we run a Friedman’s test (PRE, EXPERIMENTAL, POST) for each condition (AC, no AC) on the lips size, numbness ratings, and tactile threshold measures. As for Experiment 1, Bonferroni corrected pairwise Wilcoxon’s test was used as a post hoc test to compare the two conditions at each time point.

Second, we explored whether the increase in yes responses in 2PD across time points observed in Experiment 1 was specific to the application of AC. Therefore, we contrasted the AC and no AC conditions, using an LMM analysis with the categorical factors condition (AC or no AC), time points (PRE, EXPERIMENTAL, and POST), and distance (0, 1, 2, and 3 mm). Pseudo R2 and semipartial R2 were computed as measures of the effect sizes.

Third, we tested whether the relationship between the 2PD performance and the changes in lips size observed in Experiment 1 was a specific effect of the AC or was also observed in a control condition without AC. As in Experiment 1, we computed the difference in perceived lip size (EXPERIMENTAL-PRE) and tested the effect of this factor on the performance in the EXPERIMENTAL condition for both AC and no AC.

## Results Experiment 2

**Figure 3:**
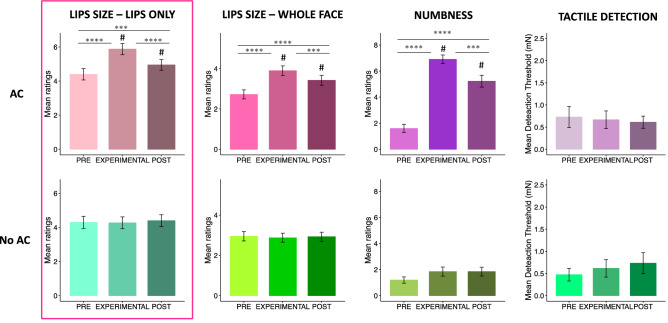
Mean rating scores for the lips size (lips only and whole face ratings), numbness and tactile detection across time points (PRE, EXPERIMENTAL, POST) of Experiment 2. The error bars indicate the standard error of the mean. **p < 0.01;***p < 0.005, ****p < 0.001; ^#^*p* < 0.01 in the difference between conditions (AC and No AC). The pink panel indicates the lip-size measure entered in the LMM analysis in the main manuscript. Results of LMM using the other lip measure are reported in the Supplementary Material.

First, we examined whether AC increased perceived lip size and changed the sensation of numbness without substantially altering tactile detection and if these effects were observed without AC. As shown in Fig. 3, we found significant changes in the AC condition for the lip size (lips only χ^2^ = 38.24, *p* < 0.001; whole face χ^2^ = 40.9, *p* < 0.001) and numbness ratings (numbness χ^2^ = 58.24, *p* < 0.001), but not for tactile detection as assessed by Semmes–Weinstein monofilament. Indeed, participants rated the size of the lips bigger and the level of numbness greater when the AC was applied compared to the other time points (*p* < 0.005 and *r* > 0.12 in all comparisons). No significant effects were observed for the no AC condition. Finally, participants rated the lips bigger and number in the AC than no AC conditions in both experimental and post time points (*p* < 0.01, *r* > 0.10 in all comparisons).

**Figure 4:**
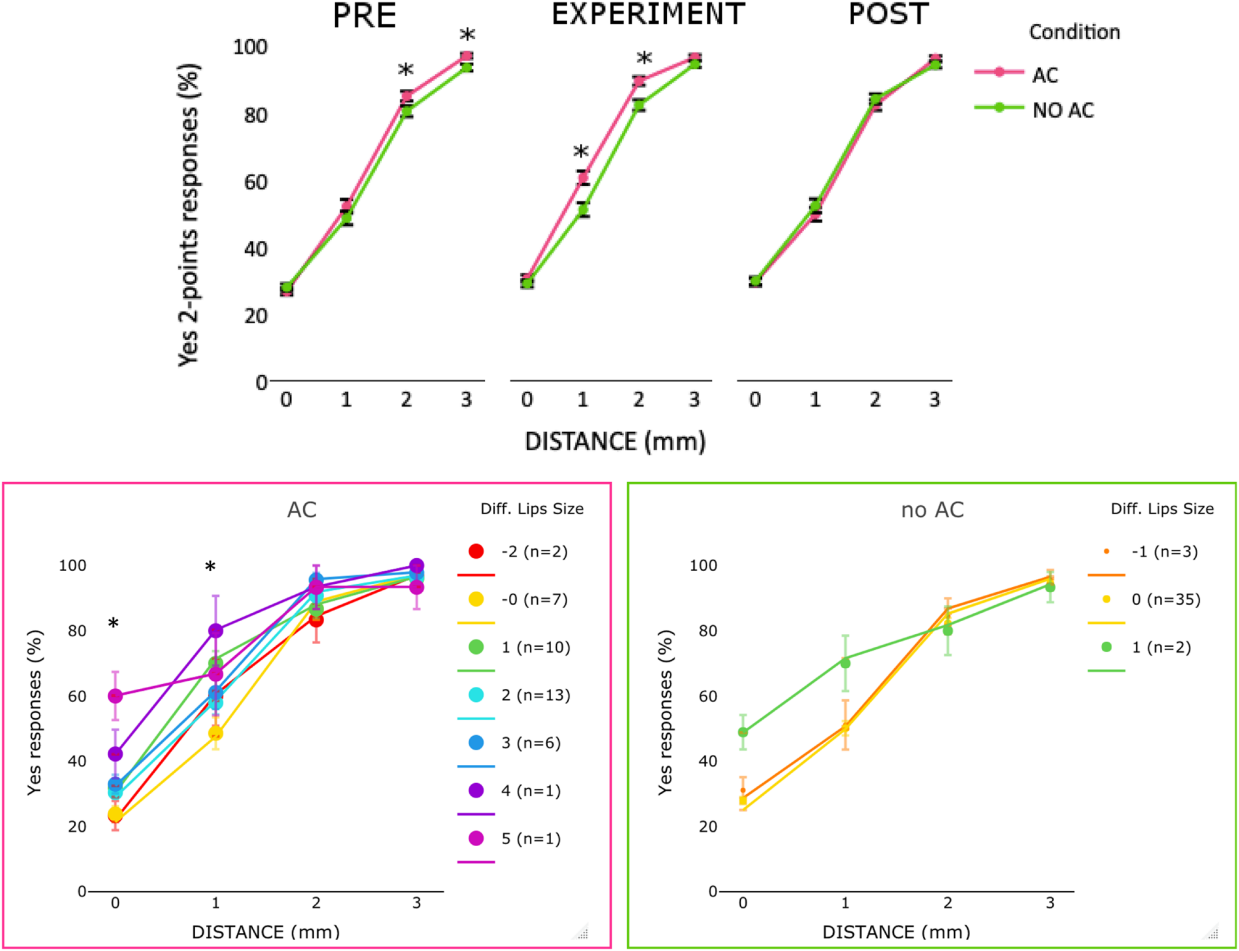
Top panel, mean (markers) and SE of the means (error bars) of *yes* responses in 2PD in PRE, EXPERIMENT and POST conditions for AC and no AC conditions at each distance (0, 1, 2, 3 mm) in Experiment 2. Bottom panel, mean (markers) and SE of the means (error bars) of *y*es responses in 2PD in AC (left panel) and no AC (right panel) conditions as a function of the distance (0, 1, 2, 3 mm) between 2 stimulation sites and the change in the perceived lip size (lips only rating) in Experiment 2. The legend describes the amount of perceived change in the lip size and the number of individuals who perceived that change. Negative numbers indicate a perceived decrease and positive numbers indicate an increase in the perceived lip size compared to PRE. In the bottom row plots, colored lines represent the estimated effects in the LMM analyses and the markers the performance of individuals in the EXPERIMENT based on change in the perceived lip size in that condition with respect to the PRE.

Second, we tested whether the effect on 2PD was restricted to the AC condition. We ran a LMM analysis with the categorical factors condition (AC or no AC), time point (PRE, EXPERIMENTAL, and POST) and distance (0, 1, 2 and 3 mm); all were included in the final model (logLik = − 10,550, χ^2^ (12) = 49.3, *p* < 0.001, pseudo R2 = 0.44) (see Supplementary Information for more details about the analysis). In line with our prediction, an improvement in 2PD was observed for the AC: performance was better after the application of AC than in the PRE condition at all distances (*z* > 2.3, *p* < 0 0.05) except for 3 mm (*z* = 0.49, *p* = 0.61). Importantly, this improvement was not observed in the no AC condition, suggesting that the observed effect with AC is not due to familiarization with 2PD. In addition, participants showed a better performance with AC than without AC at 1 mm (*z* = − 3.5, *p* < 0.001) and 2 mm (*z* = − 3.7, *p* < 0.001) distance, while similar performance between conditions was observed at 0 and 3 mm (see Fig. 4 top panel).

When looking at the response criterion, we replicated the results of Experiment 1 showing a more conservative criterion for the application of the AC at 3 mm (*M* = 0.15, *SE* = 0.01, *p* < 0.001, *r* = 0.89) as compared to the PRE condition (M = − 0.35, SE = 0.05); in this experiment, we also found a more conservative criterion for the AC at 1 mm (*M* = 0.01, *SE* = 0.01, *p* < 0.001, *r* = 0.70) than the PRE condition (*M* = − 1.04, *SE* = 0.02). However, similar effects were also observed for the no AC condition at 3 mm (PRE *M* = 0.11, *SE* = 0.05, EXPERIMENTAL *M* = 1.27, *SE* = 0.07, *p* < 0.001, *r* = 0.81), and the change in the criterion was similar for the AC and no AC condition (see Supplementary Materials).

Third, we examined whether the perceived changes in lip size influenced the yes responses in both no AC and AC conditions. Following the results of the lips only measure (see Supplementary for whole face measure). LMM confirmed our predictions that the relationship between changes in lip size and 2PD was related to the AC. For no AC condition, the final model included only the fixed factor distance (logLik = − 1768, χ^2^(3) = 1273, *p* = 0.001; pseudo R2 = 0.45), whereas for the AC condition both changes in lip size (lips only) and distance contributed to the final model (logLik = − 2429, χ^2^(6) = 4.38, *p* = 0.03; pseudo R2 = 0.45) (see Fig. 4 bottom-left panel). The main effects of distance and size were significant (see Table S1), suggesting that the yes responses increased with the distance, as well as with the perceived increase in lip size. Additionally, our prediction that the increase in lip size would be observed in trials with the greatest uncertainty was partially confirmed. There was a marginally significant increase in yes responses with the increase in the lip size at distance 1 mm (*z* = 1.9, *p* = 0.054). A significant effect was also at 0 mm (*z* = 2.3, *p* = 0.01), but not at 2 mm or 3 (*z* < 0.9, *p* > 0.39).

## Comments Experiment 2

Experiment 2 replicated the results of Experiment 1 in a larger sample of subjects and showed that the performance in 2PD with AC is not simply due to learning and/or familiarization with the task, but is a specific effect of the AC. Indeed, the increase in yes responses was observed after the application of AC but not in the control condition without AC. Instead, participants remained consistent across testing times in no AC condition. Furthermore, perceived changes in lip size were only reported and accounted for 2PD performance with the AC application. This evidence supports the hypothesis that the perceived enlargement of a body part has an effect on 2PD.

As in Experiment 1, we also found that AC induced numbness of the lips, but it did not substantially alter participants’ ability to detect tactile stimuli. One possible account of our findings is that the effects observed in Experiments 1 and 2 are non-specific effects of the application of a cream. For instance, Lévêque et al.^17^ reported an improvement in 2PD with skin hydration in older subjects. The application of the AC may have induced a certain degree of skin hydration that improved performance in the 2PD task. As previous work^33^ has shown that directing attention to a body part improves tactile discrimination, another possibility is that the results are a consequence of directing attention to the lips^34,35^. Similar mechanisms may also account for our results as the cream application may have directed participants’ attention to their lips.

To address these possible non-specific effects of AC, in Experiment 3 we examined whether the improvement in 2PD observed in Experiments 1 and 2 was linked to changes in perceived lip size induced with AC or could be observed with the application of a moisturizing cream (EucerinTM, hereafter MC). As effects of hydration on the skin have been reported^17^, we reasoned that MC could improve performance on the 2PD task; we did not, however, predict a relationship between perceived changes in lip size and performance in conditions of high uncertainty (1–2 mm) in 2PD for the MC. If AC increases the perceived size of the body and these changes modulate the improvement in the perceived distance between 2 points, one would expect a significant positive relationship between yes responses in 1 and/or 2 mm distances of 2PD and subjective enlargement of the lips.

## Methods Experiment 3

### Participants

Fourteen individuals (*M*_age_ = 22.07, *SD* = 2.7; 9 females) were tested. As for Experiment 2, power analysis with sjstats package showed that with a sample of fourteen subjects we had power of 80% (*p* < 0.05) to detect a large (*d* = 1.2) effect size to test the effect of the fixed parameters condition (AC, MC), distances (0, 1, 2, 3 mm), and time points (PRE, EXPERIMENTAL, POST).

### Task

Subjects participated in two testing sessions: in one session AC was applied to participants’ lips, while in the other session MC (EucerinTM) was applied. The structure of each session was similar to the previous experiments: 2 PD measures in 3 timepoints (PRE, EXPERIMENTAL, POST) at four distances (0, 1, 2, 3 mm). Participants were not told which cream was applied in each session and the order of sessions was counterbalanced between participants. The experimenter applying the tactile stimuli was blind to the EXPERIMENTAL condition. An electric aesthesiometer (AOS ABSOLUTE Digimatic Caliper; jaws resolution 0.01 mm) was used to test two-point discrimination. The tactile detection threshold was assessed with Semmes–Weinstein monofilament (Stoelting), and numbness was rated using a visual analog scale from 0 (no numbness) to 10 (high numbness) in the PRE, EXPERIMENTAL, and POST conditions.

### Data analysis

Dara analysis and rationale was the same as Experiment 2, with the only difference that AC was now contrasted with MC.

## Results Experiment 3

**Figure 5:**
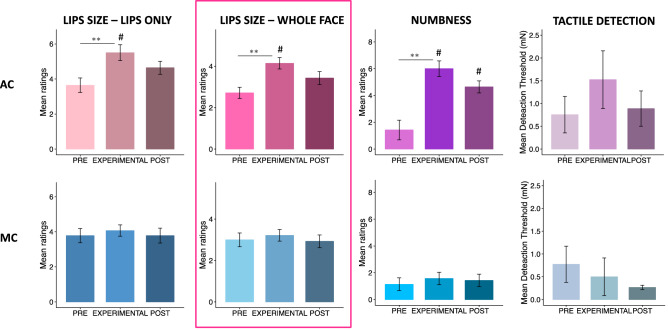
Mean rating scores for the lips size (lips only and whole face ratings), numbness and tactile detection across time points (PRE, EXPERIMENTAL, POST) of Experiment 3 for the AC (top panel) and MOIST (bottom panel). The error bars indicate the standard error of the mean. **p < 0.01; ***p < 0.005, ****p < 0.001 in the difference between time points; ^#^*p* < 0.01 in the difference between conditions (AC and MOIST). The pink panel indicates the lip-size measure entered in the LMM analysis in the main manuscript. Results of LMM using the other lip measure are reported in the Supplementary Material.

First, we tested whether AC and MC induced similar changes in perceived lip size, numbness, and tactile detection. As shown in Fig. 5, there were no significant differences for tactile detection, confirming that the anesthetization did not significantly influence tactile detection. As for Experiment 2, the perceived lip size (lips only χ^2^ = 16.57, *p* < 0.001; whole face χ^2^ = 18.89, *p* < 0.001) and numbness (χ^2^ = 14.92, *p* < 0.001) varies across time points for the AC, but no significant effects were found for the MOIST. For the lip size measure (lips only and whole face scores) and numbness participants showed higher rating in the experimental condition of the AC than of the MOIST (*p* < 0.01, *r* > 0.40).

**Figure 6:**
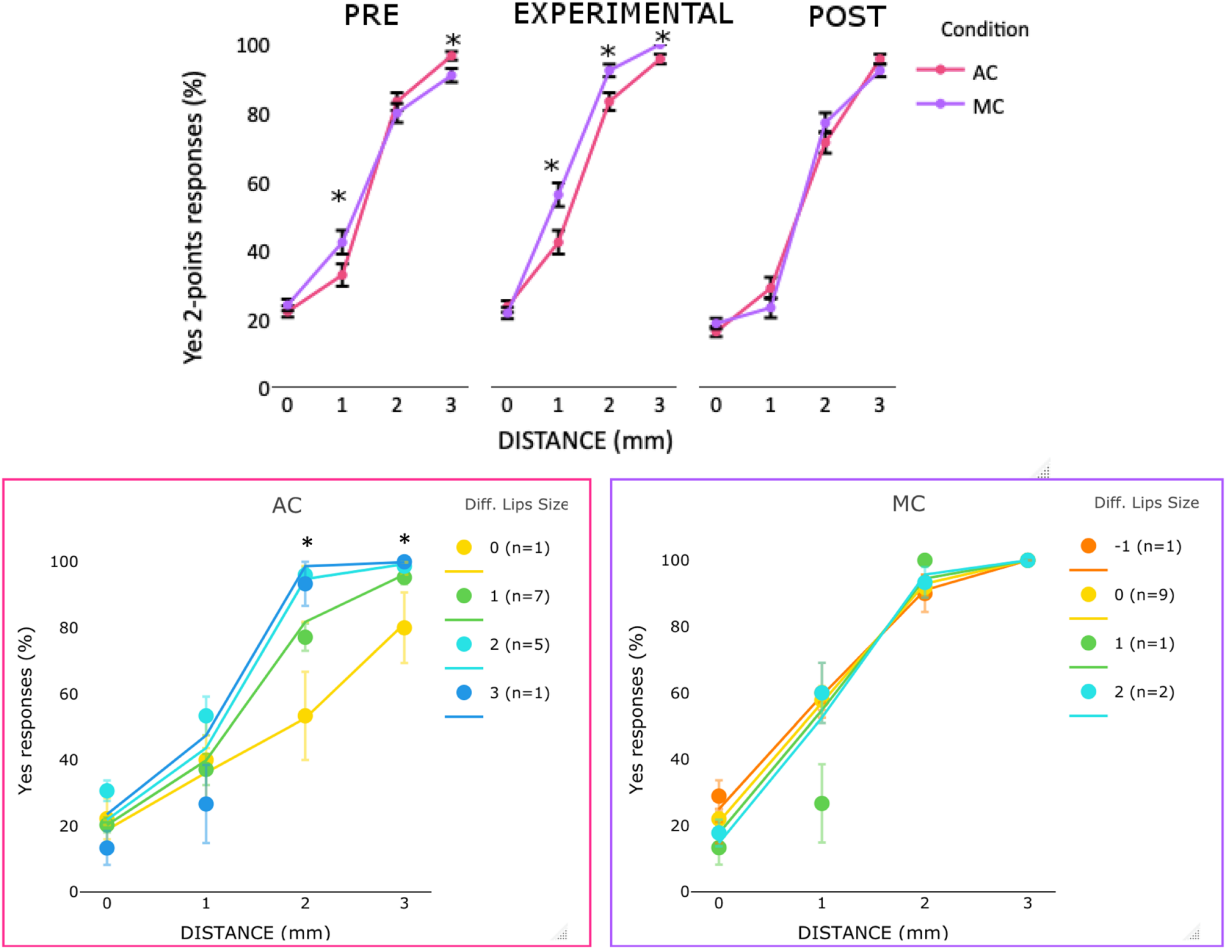
Top panel, mean (markers) and SE of the means (error bars) of yes responses in two points discrimination in PRE, EXPERIMENT and POST conditions for AC and MC at each distance (0, 1, 2, 3 mm) in Experiment 3. Bottom panel, mean and SE of the means (error bars) of *yes* responses in 2PD for AC (left panel) and MC (right panel) conditions as a function of distance (0, 1, 2, 3 mm) between 2 points and the change in the perceived lip size (whole face rating) in Experiment 3. The legend describes the amount of perceived change in the lip size and the number of individuals who perceived that change. Negative numbers indicate a perceived decrease and positive numbers indicate an increase in the perceived lip size compared to PRE. In the bottom row plots, colored lines represent the estimated effects in the LMM analyses and the markers the performance of individuals in the EXPERIMENT based on change in the perceived lip size in that condition with respect to the PRE.

Second, we investigated whether 2PD performance differed with the application of AC or MC. We ran a LMM analysis with the cream type (AC or MC), time point (PRE, EXPERIMENT, and POST) and distance (0, 1, 2 and 3 mm) as fixed categorical factors and all were included in the final model (logLik = − 3487, χ^2^(12) = 49, *p* < 0.001; pseudo R2 = 0.72) (see Table S1). As predicted, we found an increase in *yes* responses with the application of AC in the EXPERIMENTAL condition as compared to the PRE condition at 1 mm (*z* = 2.05, *p* < 0.03). The application of MC also induced an improvement at 1,2,3 mm (*z* > 2.8, *p* < 0.01 in all comparisons). In the EXPERIMENTAL condition, participants were more accurate in the MC as compared to AC at 1,2,3 mm distances (*z* > 2.8, *p* < 0.01) (see Fig. 6 top panel). Contrary to the previous experiments we did not observe significant changes in the response criterion after the application of the cream (see Supplementary Materials). Importantly, the response criterion was similar in both AC and MC.

Third, we tested whether the improvement in the performance in the EXPERIMENTAL condition was related to an increase in the perceived lip size in the MC and AC conditions. Following the results of the whole face measure (see Supplementary Materials for lips only measure). LMM analyses showed a significant contribution to the changes in lip size for the AC, but not for MC. Indeed, the model including both distance and lip size (whole face) better predicted *yes* responses than the model with only distance as a fixed factor for the AC (logLik = − 3487, χ^2^(4) = 21.7, *p* < 0.001; pseudo R2 = 0.55), but not for MC (logLik = − 517, χ^2^(3) = 702, *p* < 0.001; pseudo R2 = 0.93). Although performance was better with MC than AC at 1 and 2 mm distances, the increase in the perceived changes in lip size accounted for *yes* responses only for AC and not for the MC (see Fig. 6 bottom panels). Indeed, *yes* responses increased as a function of perceived increase in lip size only for AC, at 2 mm (*z* = 3, *p* = 0.002) and 3 mm (*z* = 2.4, *p* = 0.01) distances (see left panel Fig. 5).

## Comment Experiment 3

Experiment 3 tested whether the results of Experiments 1 and 2 were due to a non-specific effect of the AC. Consistent with previous work demonstrating an improvement in 2PD with skin hydration^17^, we observed improvement in 2PD with MC that was superior to the improvement with AC. The application of MC, however, did not induce a sensation of increased lip size and the performance on the EXPERIMENTAL condition was not associated with a perception of enlargement of the lips, as was observed for the AC. This finding speaks against demand characteristics or attentional effects as possible account of our findings with AC. One may argue that the attentional account may not be totally excluded, as the different topical effects of the two creams may have modulated the attention towards the lips to different degrees. However, if this was the case, we would expect participants’ performance to be better with AC than MC, as the alteration in sensation produced by the AC is greater than that of the MC.

The fact that a change in perceived lip size predicted *yes* responses for AC but not for MC suggests that different mechanisms may account for the effects of AC and MC on 2PD performance. As suggested by others^17^, we propose that MC increases skin volume and surface area, thereby increasing the physical distance between the skin’s mechanoreceptors. The effect of MC on 2PD would be purely bottom-up. On the other hand, the relationship between 2PD with the AC and perceived changes in lip size speaks about the role of top-down information regarding the body representation.

## General discussion

Our results demonstrate that the magnification of a body part induced by alteration of somatosensory inputs influences 2PD. The application of an AC made participants more likely to correctly discern that they had been touched at two points, particularly when the gap between the points was small (1–2 mm). Experiment 2 demonstrated that this effect was not due to learning or familiarization with the task. Experiment 3 demonstrated that a moisturizing cream improved performance on the 2PD task, a phenomenon previously described^17^. Importantly, only the AC induced the sensation of an increase in lip size and, across all three experiments, the increase in lip size accounted for the degree of improvement associated with AC in conditions of uncertainty. We suggest that the latter finding argues for different mechanisms underlying the improvement of the 2PD task with AC as compared to MC.

Our results also showed that the effects of the AC were not produced by an alteration of subjects’ response criteria or decision-making strategies. The application of the anesthetic did not induce a general bias to respond *yes* at 2 points. A more conservative criterion was observed with the application of the AC than the PRE condition at 3 mm in Experiments 1 and 2. This effect was not confirmed in Experiment 3, where the response criterion was similar in the AC and in the MC. Furthermore, in Experiment 3 changes in the response criterion were similar in the AC and no AC condition, suggesting that although participants showed an increase in the tendency to respond *no* when 2PD are presented at 3 mm distance, this response bias is not specific for the anesthetic cream.

What are the potential mechanisms behind the effects of the AC and do they differ from the MC? Although we acknowledge that AC may also serve as a moisturizer, data from Experiment 3 demonstrate that for AC—but not MC—the degree of magnification was predicted by the degree to which the cream produced a subjective enlargement of the lips, suggesting that the mechanisms underlying the effects of the AC and MC differ. Hydration of the skin increases skin volume and surface area, thereby increasing the physical distance between the skin’s mechanoreceptors^17^. Thus, if MC serves to slightly increase the distance between receptors on an “inflated” lip, this could reduce the overlap of the receptive fields of the mechanoreceptors, thereby enhancing performance.

We suggest that that a different explanation is required for the effects of the AC. Calford and Tweedale^36^ reported data from experiments in which lidocaine was used to anesthetize a digit of flying foxes or monkeys. They found that anesthetization caused an increase of the receptive fields in both contralateral and ipsilateral somatosensory cortex (Area 3b in monkeys) that was apparent within minutes and resolved over approximately 20 min. They suggested that the effect reflected an unmasking of normally silent connections that were typically inhibited by output from the denervated area; in subsequent work involving the application of capsaicin cream, a potent inhibitor of C fibers, Calford and Tweedale^37^ argued that the effect was mediated by inhibitory input from C fibers.

Gandevia & Phegan^8^ reported relevant findings in humans. They demonstrated that incomplete anesthesia of the lips produced by topical anesthetics such as lidocaine produce a substantial increase in the perceived size of the lips that was observed within minutes. As lidocaine is also known to inhibit C-fibers^38^, they proposed that the subjective increase in lip size was attributable to by a loss of inhibitory C-fibers input with resultant increase in receptive field size in S1 and an increased responsiveness of neurons subserving both the anesthetized and surrounding areas to respond to what would typically be subthreshold inputs. One possible account of the enhanced performance on our 2PD task, therefore, is that lidocaine inhibits C fiber inputs with a resultant increase in receptor field size of neurons in S1. The increase in receptive field size could result in a larger overlap across receptive fields, a similar principle as coarse coding^39^. In this condition, a single tactile stimulus would fall within the receptive fields of multiple neurons. As the region of the skin subserved by the intersection of multiple mechanoreceptors is likely to be smaller than the area subserved by a single neuron in the absence of anesthetic, the site of the touch may be marked with greater precision with AC, thereby enhancing the ability to distinguish between one and two points. We speculate that this account might also explain a seemingly paradoxical finding in the AC condition – that is, poorer performance on one-point trials. After the application of AC, each stimulus would fall inside more receptive fields, inducing a response from a larger number of neurons than in the normal condition. An increase in the number of neurons responding to a single touch may, on one-point stimulation trials, lead to a bias to perceive that two sites were stimulated. It must be acknowledged, however, that a potential objection to this account is that the data supporting the effects of capsaicin and topical anesthetics on C fiber function come from animal studies in which the agents are injected rather than applied topically.

An alternative, but not mutually exclusive interpretation, is that changes in 2PD reflect the top-down effect of changes in the body representation induced by alterations in somatosensory input. That is, the perceived change in body size may be analogous to the effects on somatosensory^4,5^ and motor^3,10^ cortex function induced by alterations in visual input. Previous work has demonstrated that accuracy in 2PD measured on the arms increases when looking at this body part with magnifying lenses^4^ We also demonstrated that magnification of the visual image of one’s hand increases the motor cortex excitability and induces a rapid increase of the cortical representation of this body part in M1^11^ and improves motor function in patients with stroke^10,12^.

Taylor-Clarke et al.^7^ argued that the effects of magnification of the visual image of the image of hand on somatosensory function were mediated by the parietal cortex. Similarly, to account for our data on the effects of magnification on motor function, we suggested that visual magnification of a body part induced changes in M1^12^ and proposed that these effects might be mediated by multimodal areas of the parietal cortex^5,7,40^ that code the size and shape of the individual’s body. We have previously argued for multiple representations of the body^3^, including what we termed the body form representation that is assumed to code the size and shape of an individual’s body on the basis of online, constantly updated multi-motor sensory information.

We suggest that the effects of magnification on 2PD caused by AC are induced by changes in the representation of the lips in S1 that is densely and richly interconnected with multimodal parietal areas such as the anterior intraparietal sulcus and secondary somatosensory regions in the parietal operculum (SII). Of relevance in this setting, Konen et al.^40^ demonstrated that the anterior intraparietal sulcus mediates the beneficial effect of vision of the limb in tactile discrimination. We assume that the changes in S1 quickly and reversibly alter multimodal parietal cortices that support, at least in part, the body form representation that is interrogated when subjects are asked to make explicit judgments about their body in the 2PD task. Although speculative, we believe this account of the genesis of the AC-induced magnification effects to be consistent with prior work.

We note that participants’ baseline performance on 2PD was very different across experiments. Several factors could account for this difference. One potential issue is that the tools and procedures used to test two-point discrimination (standard and digital caliper) differed slightly across (but not within) experiments. A second factor is that there are individual differences in tactile perception. However, we note that the effects observed remained when controlling for baseline accuracy, suggesting that these factors could not explain our results. Furthermore, the present study tested the effect of the increase in the perceived size induced in the somatosensory modality focusing on a specific body part, the lips. Similar results could be obtained by testing other body parts, and future studies should test this hypothesis directly.

This study has limitations. First, the limited number of between point distances assessed (0, 1, 2, 3 mms) constrained the analyses that could be performed. Second, we did not control the amount of AC cream applied. Future work should explore the relationship between the amount of AC applied, numbness ratings and behavior. Third, the use of lips size judgment tools depicting a Caucasian woman may have introduced a bias on the part of participants. Future work should use more neutral lips size ratings and test the possible effects of race and sex on lips size judgment and 2PD performance. Fourth, we did not investigate whether the application of AC induced changes in the skin temperature. As 2PD performance changes with the skin temperature^41^, it is possible that changes in skin temperature with the AC may have influenced participants’ 2PD. Fifth, in Experiments 2 and 3, numbness ratings did not return to baseline in the POST condition; whether residual effects of the AC were relevant to behavior remains uncertain and should be systematically explored. Finally, information regarding the use of cosmetics or cocoa butter as well as environmental factors that may influence the level of lip hydration was not collected in the study. It is possible that the difference in 2PD performance in the PRE condition of both Experiments 2 and 3 may be due to a different level of lips hydration prior to starting the experiment.

To conclude, our data showed that the magnification of a body part derived from somatosensory inputs also has a beneficial effect on tactile perception. Although the mechanisms underlying the improvement in 2PD after AC remain speculative, a major contribution of the present work is to show that the body representation is dynamic and may be manipulated in minutes by altering sensory input**.**

## Supplementary Information

Supplementary Information.

## Data Availability

The datasets generated and/or analysed during the current study are available in OSF the repository, at this link https://osf.io/mhtka/.
