# Supplementary material for: Apparent increase in lip size influences two-point discrimination
Source: Sci Rep. 2023 Feb 22;13:3082. doi: 10.1038/s41598-023-30067-3 (PMC9947174; doi:10.1038/s41598-023-30067-3)
Supplement: Supplementary file 1 — Supplementary Information. [file 41598_2023_30067_MOESM1_ESM.docx]

**Supplementary Information**

**Results Experiment 1**

*Analysis 1 Effect of AC:* *Comparison with the PRE-POST condition*

The percentage of yes responses was similar in PRE and POST conditions, except for 2 (*z*=4.7, *p*<0.001) and 3mm (*z*=2.4, *p*=0.01).

*Analysis 1 Effect of AC for Response Criterion*

Results of the response criterion are reported in SI Figure 1 and showed an effect of the timepoints at the 3 mm distances condition. Wilcoxon’s test showed that the only comparisons surviving Bonferroni’s Correction was the comparison between the experimental and the PRE condition (*p*=0.002, *r*=0.60)

**SI Figure 1:** Mean (SEM) of the criterion for each timepoints and distances

*Analysis 2* *Effect of lip size: effect for the POST condition*

For the POST condition, the difference in the lips size did not contribute to the model fit for whole face (logLik= -706, χ^2^ (4)=2.2, *p*=0.68; pseudo R2=0.54) measure.

*Analysis 2* *Effect of lip size: lips only rating*

The LLM analysis exploring the effect of the perceived lips size was also carried out on the difference in the lips size based on the lips only rating and showed similar results as the whole face rating. The effect of the difference in lips size contributed significantly to the model fit for the EXPERIMENTAL condition (logLik= -684, χ^2^ (4)=16.5, *p*=0.002; pseudo R2=0.57) (see SI Figure 2) and yes responses increased as function of the perceived changes in lips size at 1,2, and 3 mm (in all comparisons *z*>2.8, *p*<0.01). For the POST too the difference in lips size contributed significantly to the model fit for yes responses (logLik= -702, χ^2^ (4)=10, *p*=0.03; pseudo R2=0.54), but post hoc test did not show any significant effect of the change in the lips size for any of the distances.

**SI Figure 2:** Mean (markers) and SE of the means (error bars) of yes responses in two points discrimination in AC (left panel) and MC (right panel) conditions as function of the distance (0,1,2,3 mm) between 2 points and the change in the perceived lips size (lips only rating) in Experiment 1. The different size/coloring of the markers described the performance of individuals based on change in the perceived lips size in that condition with respect to the PRE. The legend describes the amount of perceived change in the lip size and the number of individuals who perceived that change. Negative numbers indicate a perceived decrease and positive numbers indicate an increase in the perceived lip size compared to PRE. In every plot, colored lines represent the estimated effects in the LMM analyses.

**Table S1.** Omnibus of logistic mixed effect model analysis.

|  | Analysis 1: Effect of AC | Analysis 2: Effect of the perceived lips (lips only) | Analysis 2: Effect of the perceived lips (whole face) |
| --- | --- | --- | --- |
| **EXPERIMENT 1** | **Time:** F=0.41, p=0.6, semi-partial R2= 0.29  **Distance:** F=449, p<0.001, semi-partial R2= 0.99  **Time x Distance:** F=8.07, p<0.001, semi-partial R2=0.89 | **EXPERIMENTAL:**  **Lips size:** F=4.9, p=0.02, semi-partial R2= 0.83  **Distance:** F=482, p<0.001, semi-partial R2= 0.99  **Lips size x Distance:** F=12.1, p=0.007, semi-partial R2=0.80.  Increase in yes as function of size for 1,2,3, mm (z>2.3, p<0.05)  **POST:**  **Lips size:** F=0.39, p=0.53, semi-partial R2= 0.31  **Distance:** F=158, p<0.001, semi-partial R2= 0.99  **Lips size x Distance:** F=3.2, p=0.02, semi-partial R2=0.76  The size did not reach significance for any distance | **EXPERIMENTAL:**  **Lips size:** F=9.6, p=0.001, semi-partial R2= 0.90  **Distance:** F=166, p<0.001, semi-partial R2= 0.99  **Lips size x Distance:** F=0.4, p=0.74, semi-partial R2=029  Increase in yes as function of size for 1 (z=3.1, p=0.001) 2, mm (z=2.7, p=0.02)  **POST:**  **Lips size:** F=0.16, p=0.16, semi-partial R2= 0.14  **Distance:** F=164, p<0.001, semi-partial R2= 0.99  **Lips size x Distance:** F=0.70, p=0.02, semi-partial R2=0.41 |
| **EXPERIMENT 2** | **Time:** F=2.3, p=0.09, semi-partial R2= 0.70  **Distance:** F=1491, p<0.001, semi-partial R2= 0.99  **Condition:** F=6.9, p=0.008, semi-partial R2= 0.87  **Time x Distance:** F=0.90, p=0.48, semi-partial R2= 0.47  **Time x Cream** F=5.4, p=0.003, semi-partial R2= 0.84  **Distance x Cream** F=5.8, p<0.001, semi-partial R2= 0.85  **Time x Distance x Cream:** F=2.0, p=0.054, semi-partial R2=0.67 | **AC:**  **Lips size:** F=4.4, p=0.03, semi-partial R2= 0.81  **Distance:** F=256, p<0.001, semi-partial R2= 0.99  **Lips size x Distance:** F=1.0, p=0.36, semi-partial R2=0.51.  **No AC**  **Lips size:** F=0.42, p=0.51, semi-partial R2= 0.30  **Distance:** F=254, p<0.001, semi-partial R2= 0.99  **Lips size x Distance:** F=1, p=0.36, semi-partial R2=0.51. | **AC:**  **Lips size:** F=2.1, p=0.14, semi-partial R2= 0.68  **Distance:** F=256, p<0.001, semi-partial R2= 0.99  **Lips size x Distance:** F=0.34, p=0.79, semi-partial R2=0.26  **No AC**  **Lips size:** F=0.42, p=0.83, semi-partial R2= 0.04  **Distance:** F=254, p<0.001, semi-partial R2= 0.99  **Lips size x Distance:** F=0.79, p=0.49, semi-partial R2=0.44 |
| **EXPERIMENT 3** | **Time:** F=4.1, p=0.01, semi-partial R2= 0.80  **Distance:** F=576, p<0.001, semi-partial R2= 0.99  **Condition:** F=2.8, p=0.09, semi-partial R2= 0.73  **Time x Distance:** F=3.8, p=0.008, semi-partial R2= 0.79  **Time x Cream** F=0.53, p=0.58, semi-partial R2= 0.34  **Distance x Cream** F=3.8, p=0.008, semi-partial R2= 0.79  **Time x Distance x Cream:** F=3.2, p=0.003, semi-partial R2=0.76 | **AC:**  **Lips size:** F=0.14, p=0.70, semi-partial R2= 012  **Distance:** F=87, p<0.001, semi-partial R2= 0.99  **Lips size x Distance:** F=6, p<0.001, semi-partial R2=0.85.  **MC**  **Lips size:** F=0.02, p=0.87, semi-partial R2= 0.02  **Distance:** F=77.6, p<0.001, semi-partial R2= 0.98  **Lips size x Distance:** F=0.1, p=0.93, semi-partial R2=0.11. | **AC:**  **Lips size:** F=0.78, p=0.37, semi-partial R2= 044  **Distance:** F=87, p<0.001, semi-partial R2= 0.98  **Lips size x Distance:** F=5.5, p<0.001, semi-partial R2=0.44.  **MC**  **Lips size:** F=0.60, p=0.43, semi-partial R2= 0.02  **Distance:** F=76.4 p<0.001, semi-partial R2= 0.98  **Lips size x Distance:** F=0.58, p=0.62, semi-partial R2=0.11. |

**Results Experiment 2**

*Analysis 1 Effect of AC*

The LLM analysis with condition (AC or no AC), time point (PRE, EXPERIMENTAL, and POST) and distance (0, 1, 2 and 3 mm) showed an effect of the condition in the PRE. Indeed, performance in the AC was better than the no AC at 2mm (*z*=2.1, *p*=0.03) and 3 mm (*z*=2.9, *p*=0.03); while no significant differences between these two conditions were observed in the POST. To test if the performance at baseline could account for the observed difference in the EXPERIMENTAL condition, we carried out an additional LMM analysis on this condition inserting the *yes* responses at baselines as a fixed factor. This factor did not improve the model fit with respect to the model with distance and condition as fixed factor (logLik= -3534, χ^2^ (8)=10.8, *p*=0.20; pseudo R2=0.44) and its inclusion did not change our results, suggesting that the variability in the baseline performance across condition could not account for the difference observed in the EXPERIMENTAL condition. Furthermore, for the AC, performance in the EXPERIMENTAL condition was better than the POST for 1 (*z*=4, *p*<0.001) and 2 (*z*=3.6, *p*<0.001) mm; no significant differences were observed between POST and EXP. For the No AC, performance was consistent across time points for all the distances.

*Analysis 1 Effect of AC for Response Criterion*

The criterion differed in the AC than in the No AC across all time points and distances (p<0.001 in all comparisons) (see SI Figure3). To explore these results further, we computed the difference in the criterion between the EXPERIMENTAL and PRE condition. This difference was not significant for any of the distances, suggesting a similar change in criterion with and without the cream application.

**

**SI Figure 3:** Mean (SEM) of the criterion for each timepoints and distances for the AC and No AC conditions.

*Analysis 2* *Effect of lip size: whole face rating*

The LLM analysis exploring the effect of the perceived lips size was also carried out on the difference in the lips size based in the whole face rating. The effect of the difference in lips size did not contribute significantly the model fit (logLik= -1735, χ^2^ (4)=2, *p*=0.14; pseudo R2=0.45) (SI Figure 4). This was also true for the no AC condition (logLik= -1767, χ^2^ (4)=2.5, *p*=0.64; pseudo R2=0.45).

**SI Figure 4:** Mean (markers) and SE of the means (error bars) of yes responses in two points discrimination in AC (left panel) and MC (right panel) conditions as function of the distance (0,1,2,3 mm) between 2 points and the change in the perceived lips size (whole face rating) in Experiment 2. The different size/coloring of the markers described the performance of individuals based on change in the perceived lips size in that condition with respect to the PRE. The legend describes the amount of perceived change in the lip size and the number of individuals who perceived that change. Negative numbers indicate a perceived decrease and positive numbers indicate an increase in the perceived lip size compared to PRE. In every plot, colored lines represent the estimated effects in the LMM analyses.

**Results Experiment 3**

*Analysis 1 Effect of AC*

The LLM analysis with condition (AC or no AC), time point (PRE, EXPERIMENTAL, and POST) and distance (0, 1, 2 and 3 mm) showed an effect of the condition in the PRE. The percentage of *yes* responses was higher in MC than AC at 1mm (*z*=2, *p*=0.04) and in the AC than MC at 3mm (*z*=2.3, *p*=0.01) (see SI Figure 5). As in Experiment 2, we examined if the performance in the PRE conditions could account for the results observed in the EXPERIMENTAL and POST condition; to address this point, we ran a LMM on accuracy in these two conditions and tested the effect of baseline accuracy. This factor did not contribute significantly to the model fit and its inclusion did not change our results. No significant difference between AC and MC was observed in the POST.

*Analysis 1 Effect of AC for Response Criterion*

Response criterion did not change across timepoints for either AC or MC and the differences in the response criterion between the AC and MC did not reached level of significance.

**SI Figure 5:** Mean (SEM) of the criterion for each timepoints and distances for the AC and MC conditions.

*Analysis 2* *Effect of lip size: lips only rating*

The LLM analysis exploring the effect of the perceived lips size was also carried out on the difference in the lips size based on the lips only rating. The effect of the difference in lips size contributed to the model fit of yes responses for AC (logLik= -567, χ^2^ (4)=19, *p*<0.001; pseudo R2=0.55), but not for MC (logLik= -517, χ^2^ (4)=0.4, *p*=0.9) (see SI Figure 6). *Yes* responses increased as a function of perceived increase in lip size only for AC, at 3 mm (*z*=2.4, *p*=0.01).

**SI Figure 6:** Mean (markers) and SE of the means (error bars) of yes responses in two points discrimination in AC (left panel) and MC (right panel) conditions as function of the distance (0,1,2,3 mm) between 2 points and the change in the perceived lips size (lips only face rating) in Experiment 3.. In every plot, colored lines represent the estimated effects in the LMM analyses and the markers the performance of individuals in the EXPERIMENT based on change in the perceived lip size in that condition with respect to the PRE. Negative numbers indicate a perceived decrease and positive numbers indicate an increase in the perceived lip size compared to PRE.
